# Usefulness of the Hospital Information System for maternal mortality surveillance in Brazil

**DOI:** 10.1590/1980-549720230007.2

**Published:** 2023-01-09

**Authors:** Olívia Tavares Ranzani, Maria de Fátima Marinho, Ana Luiza Bierrenbach

**Affiliations:** I Instituto de Ensino e Pesquisa do Hospital Sírio-Libanês – São Paulo (SP), Brasil.; II Vital Strategies – São Paulo (SP), Brasil.

**Keywords:** Maternal mortality, Hospital information systems, Information systems, Surveillance, Death, Brazil, Mortalidade materna, Sistema de informação hospitalar, Sistemas de informação, Vigilância, Óbito, Brasil

## Abstract

**Objective:**

To evaluate the capability of hospital records in the Hospital Information System (SIH) to add valuable and complementary information to the Mortality Information System (SIM) in studies on maternal mortality. We calculated and compared the maternal mortality ratio from the SIH and SIM databases, by age group and region, to highlight differences between groups and assess the coverage of maternal deaths using SIH compared with SIM.

**Methods:**

Obstetric hospitalizations were defined based on three sources (codes ICD-10 in diagnoses; procedures; billing information). Hospital and SIM mortality ratios were calculated by dividing maternal deaths in hospitals affiliated to the Unified Brazilian Health System (SUS) per live births (SINASC) in the same hospitals.

**Results:**

In 2019, we identified 2,497,957 obstetric admissions, 0.04% (946) with in-hospital mortality as outcome. The presence of three criteria identified 98% of obstetric hospitalizations and 83% of obstetric hospitalizations with death as outcome. The comparison of mortality ratios between SIH (45.5 MMR; 95%CI 42.7 – 48.5) and SIM (49.7 MMR; 95%CI 46.7 – 52.8) was not statistically significant (p-value: 0.053).

**Conclusion:**

The analysis of SIH was able to provide additional information for the monitoring and surveillance of maternal health in Brazil. Although there are differences between the mortality rates, the SIH, as a complementary information system to the SIM, may be valid in studies on maternal mortality and morbidity.

## RESUMO

**Objetivo:**

Avaliar a capacidade dos registros hospitalares (SIH) em adicionar informações úteis e complementares ao Sistema de Informações sobre Mortalidade (SIM) no entendimento da mortalidade materna. Calcular e comparar a Razão de Mortalidade Materna (RMM) hospitalar e a RMM do SIM dos óbitos maternos ocorridos em hospitais, por faixa etária e por região, para demonstrar diferenças entre os grupos e avaliar a cobertura de óbitos maternos do SIM em relação ao SIH.

**Métodos:**

As internações obstétricas foram definidas com base em três critérios (códigos da 10^a^ Revisão da Classificação Estatística Internacional de Doenças e Problemas Relacionados à Saúde — CID-10 nos diagnósticos; procedimentos; cobrança de parto). As RMM hospitalar e do SIM foram calculadas dividindo-se os óbitos maternos ocorridos nos hospitais conveniados ao Sistema Único de Saúde (SUS) pelos nascidos vivos (Sistema de Informação sobre Nascidos Vivos — SINASC) desses estabelecimentos.

**Resultados:**

Em 2019, identificamos 2.497.957 registros de internações obstétricas, 0,04% (946) com óbito hospitalar. Os três critérios localizaram 98% das internações obstétricas e 83% das internações com óbitos, revelando inconsistências entre diagnósticos e procedimentos. A comparação entre a RMM do SIH (45,5, intervalo de confiança — IC95%, 42,7–48,5) e a do SIM (49,7, IC95%, 46,7–52,8) não foi estatisticamente significante (p-valor 0,053).

**Conclusão:**

A análise do SIH foi capaz de prover informações adicionais ao monitoramento e vigilância da saúde materna no Brasil. Embora haja diferenças entre as RMM, o SIH como sistema de informação complementar ao SIM pode ser válido nos estudos sobre mortalidade e morbidade materna.

## INTRODUCTION

Maternal mortality (MM) reflects the access to and quality of health services in a country, as it is considered preventable in most situations. It is synonymous with neglect of women’s human rights and lack of attention to sexual and reproductive health^1,2^.

The standard calculation of Maternal Mortality Ratio (MMR) in Brazil uses maternal deaths from the Mortality Information System (SIM) as numerator, fed by the Death Certificate (DC) and corrected by an active search for deaths^3^. Surveillance of deaths of women of childbearing age (WCA), mandatory since 2008^4^, seeks to understand possible failures of the health system by not preventing deaths, in the fight against under-reporting, solving possible failures in the registration of deaths classified as erroneously, cases without a DC, and cases not registered in the SIM^3,5,6^.

There are uncertainties about the MM burden across the world, mainly due to the lack of robust data, precisely in low-income countries where estimates are higher. Over the past few decades, efforts to measure MM have been systematically and globally made. Several initiatives have been undertaken, such as the incorporation of new ways of capturing data on maternal deaths to existing sources, the development of new analytical tools and approaches, and the training of responsible personnel in the countries to correctly interpret data obtained. The choice of one method over another depends on the characteristics of the population being investigated, on data sources and on human and financial resources available to measure deaths. However, studies have shown that these methods have limitations and, whenever possible, the use of official civil records should be extended for a closer determination of maternal deaths and cases of WCA affected by severe obstetric complications^7–10^.

In this study, we evaluated the use of the Hospital Information System (SIH) as a complementary surveillance tool to the SIM, selecting records of hospital admissions related to pregnancy and the puerperium of WCA. We believe that data from SIH bring additional information to the field. Finally, we calculated the hospital MMR and compared to the MMR calculated using the SIM.

## METHODS

### Study Design

This is a cross-sectional observational study based on records of hospital admissions of WCA in the SIH, all occurring in 2019, across Brazil. The population sample included women aged 10 to 49 years who had been hospitalized for obstetric causes^11^.

### Databases

Open access data were obtained from the Department of Informatics of the Unified Health System (DATASUS), of the Ministry of Health. To compare with the standard calculation, the numerator of the MMR was extracted from the SIM. For the denominator of overall MMR, data were extracted from the Information System on Live Births (SINASC).

### Database construction

After extracting the 2019’s data from SIH, the following records were excluded:

1. Long-term hospitalization (variable IDENT=5);
2. Males;
3. Females under the age of 10 and over 49 years old.

Admissions of WCA (10–49 years old) were sorted into two groups: admissions for obstetric causes and admissions for other causes.

Hospital admissions for obstetric causes were defined as presence of at least one obstetric criterion among the variables:

1. Diagnoses (main and secondary) of reason for hospitalization;
2. Procedure performed; and
3. Billing discharge coding for obstetric/maternal hospitalization.

The definition was classified as follows:

1. Obstetric/maternal diagnoses: main and secondary diagnoses of cause of hospitalization with the codes of the 10th Revision of the International Classification of Diseases and Related Health Problems — ICD-10^12^ in Chapter XV (Pregnancy, Childbirth and Puerperium), except codes O96 (late maternal death) and O97 (death due to direct obstetric sequelae), as they do not include the period of up to 42 days after delivery, according to the classic definition of maternal death^1,3^. The following codes from other chapters were included: A34 (obstetric tetanus), D39.2 (malignant hydatidiform mole), F53 (mental and behavioral disorders associated with the puerperium), and M83.0 (puerperal osteomalacia)^3^.
  Hospitalizations with diagnoses of postpartum pituitary necrosis (E23.0), when no other indicator validated that it was an obstetric hospitalization (e.g., another primary and/or secondary diagnosis), were not included in the sample, as there are other causes of pituitary necrosis beyond the postpartum period. We used the same criterion for hospitalizations with diagnoses of diseases caused by the human immunodeficiency virus (B20 to B24).
2. Obstetric/maternal procedures: according to the Management System of Procedures, Medications, Orthoses, Prostheses and Special Materials of the Unified Health System (SIGTAP/SUS)^13^, the following were selected: treatment of clinical intercurrences during pregnancy (303100044); normal delivery (310010039); normal delivery at a normal delivery center (CPN) (310010055); normal delivery in high-risk pregnant women and/or eclampsia (310010047); cesarean delivery (411010034); cesarean delivery with tubal ligation with diagnoses of sterilization (411010042); cesarean delivery in high-risk pregnant women (411010026); treatment of edema, proteinuria and hypertensive disorders in pregnancy, childbirth and puerperium (303100036); treatment of eclampsia (303100028); treatment of other maternal disorders predominantly related to pregnancy (411020056); treatment of complications predominantly related to the puerperium (303100010); surgical treatment of acute postpartum uterine inversion (411010085); manual reduction of postpartum acute uterine inversion (411010050); puerperal hysterectomy (411020030); manual placental abruption (411010018); suture of pelvic path lacerations (in childbirth before admission) (411010077); postpartum episiotomy resuture (411010069); post-abortion uterine evacuation by manual intrauterine aspiration (MIVA) (409060070); post-abortion/puerperal curettage (411020013); surgicalz treatment of ectopic pregnancy (411020048); treatment of hydatidiform mole (molar pregnancy without childbirth) (303100052); uterine curettage of hydatidiform mole (409060054); embryotomy (411020021); cervical cerclage (409060011).
3. Billing discharge coding for obstetric/maternal hospitalization: reasons for billing ascertained at hospital discharge due to delivery procedure, divided into seven categories according to the SIH^11^: discharge of the mother/postpartum woman and newborn (6.1), discharge of the mother/postpartum woman and stay of the newborn (6.2), discharge of the mother/postpartum woman and death of the newborn (6.3), discharge of the mother/postpartum woman with fetal death (6.4), death of the pregnant woman and the conceptus (6.5), death of the mother/postpartum woman and discharge of the newborn (6.6), death of the mother/postpartum woman and stay of the newborn (6.7).

### Maternal Deaths in SIM

We obtained two MMR out of SIM data: 1) overall MMR, which corresponds to the crude MMR as the Ministry of Health (without correction factor); 2) Adapted RMM, with deaths selected from SIM with inclusion criteria similar to those of SIH.

For the overall MMR, we selected SIM deaths according to the Ministry of Health criteria available in the 2019 technical note “Deaths of women of childbearing age and maternal deaths”^14^. The latter were allocated according to place of residence, regardless of age and prioritization of underlying cause of death, even in cases of inconsistency between declared maternal cause and time of death (pregnancy-puerperal cycle, from 43 days to one year after delivery or outside these periods). The cause of death was based on ICD-10^12^, including codes from Chapter XV (Pregnancy, Childbirth and Puerperium) (except deaths outside the puerperal pregnancy cycle—codes O96 and O97) and codes from other chapters:

1. B20 to B24 (diseases caused by the human immunodeficiency virus); D39.2 (malignant hydatidiform mole); E23.0 (postpartum pituitary necrosis)—provided the woman was pregnant at the time of death or had been pregnant up to 42 days before death;
2. A34 (obstetric tetanus); F53 (mental and behavioral disorders associated with the puerperium); M83.0 (puerperal osteomalacia)—death occurring within 42 days after end of pregnancy or cases without information on the time elapsed between end of pregnancy and death.

In this study, we used the SIM variable “TPMORTEOCO” to separate deaths per time of occurrence in relation to delivery time. The variable has seven options:

1. Pregnancy;
2. Childbirth;
3. Abortion/Miscarriage;
4. Up to 42 days after delivery;
5. From 43 days to 1 year after delivery;
6. Not in these periods; and
7. Ignored.

For the adapted MMR, we used the following criteria:

1. Deaths occurred in the timeframe associated with the moment of death, defined according to the variable “TPMORTEOCO”, excluding those that occurred “between 43 days and 1 year after delivery”; or that “did not occur in these periods”, “ignored” and unfilled;
2. Deaths occurred in a SUS-affiliated hospital, thus excluding maternal deaths that did not occur in hospitals and selecting the same SUS-affiliated health establishments contained in the SIH data of 2019.

For this, the variable that identifies the health facility (variable “CNES” in SIH and “CODESTAB” in SIM) were used and paired. As the SIH had all obstetric hospitalizations, all values of “CNES” were selected, regardless of the outcome death. Then, we verified which institutions related to obstetric hospitalizations of the SIH were present in the SIM. SIM maternal deaths that did not occur in health facilities listed in the 2019 SIH obstetric admissions were excluded for comparison purposes between both systems. The same criterion for selection of institutions (hospitals and health facilities) was used to select births in the SINASC during 2019, to calculate the number of live births (LB) in hospitals affiliated with SUS, based on obstetric admissions identified in the SIH in the same year by using the variable “CNES” in the SIH and “CODESTAB” in the SINASC.

In summary, we calculated:

1. Hospital MMR: deaths identified in the SIH based on presence of at least one of the three criteria (diagnosis, procedure and billing); the denominator was LB in hospitals and health institutions affiliated with the SUS and present in the SIH in 2019.
2. SIM adapted MMR: maternal deaths that occurred in the same hospitals and health facilities found as obstetric admissions in the SIH; the denominator was the same number used to calculate the hospital MMR.
3. SIM overall MMR: maternal deaths per total number of LBs, regardless of the place of death and birth.

According to the case selection methodology in both SIH and SIM, there were no missing data.

### Statistical Analysis

Categorical variables were compared using the Pearson’s χ² test or the Fisher’s exact test, while continuous variables were described as means (standard deviation) or medians (p25–p75) and compared using the Student’s t test or Wilcoxon-Mann-Whitney’s test, as appropriate. In addition to the nationwide assessment, we also assessed the five macro regions and age categories.

For all analyses, values of p<0.05 were considered significant. Database management and statistical analyses were performed using the Stata-15 software (Statacorp, College Station, Texas, USA).

The study was approved by the Research Ethics Committee of the Teaching and Research Institute of Hospital Sírio Libanês (CAAE 34126820.0.0000.5461/opinion number 4.151.968) on July 13, 2020. The Free Informed Consent Form was not required, as the study used secondary data and researchers had no contact with people/patients.

## RESULTS

**Figure 1.**
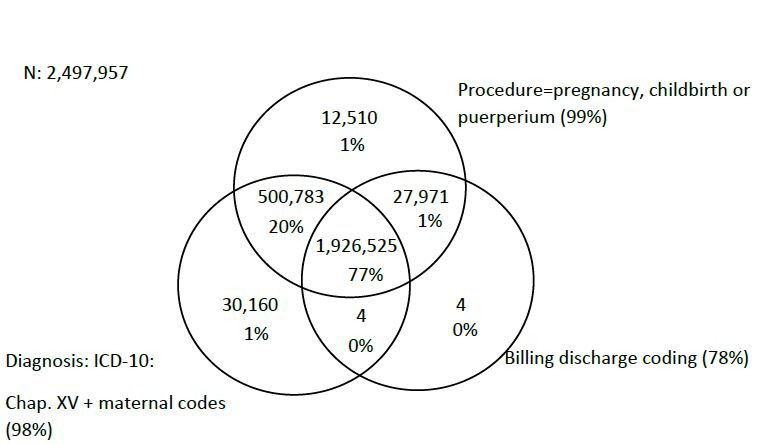
Venn Diagram of obstetric hospitalizations of women of childbearing age by hospitalization diagnoses, procedures performed and delivery charge according to the Hospital Information System’s records. Brazil, 2019.

**Figure 2.**
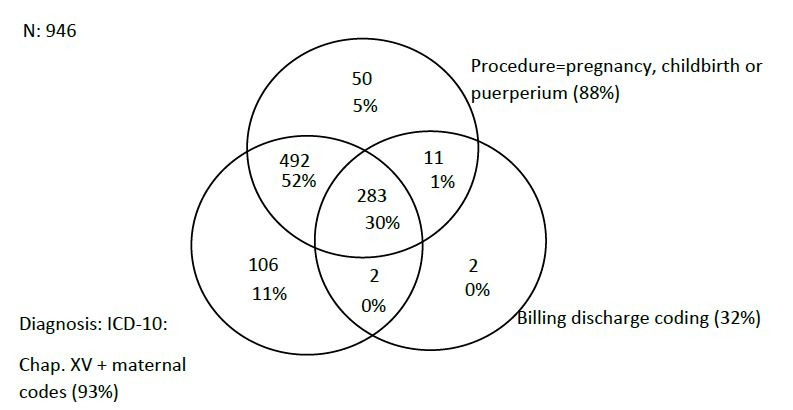
Venn diagram of obstetric hospitalizations with deaths of women of childbearing age by hospitalization diagnoses, procedures performed and delivery charge according to the Hospital Information System’s records. Brazil, 2019.

In 2019, 4,236,387 WCA were hospitalized in SUS-affiliated hospitals in the country. Of these, 2,497,957 (59%) had obstetric causes and 946 (0.04%) had maternal death as outcome. As for criteria agreement (1. diagnoses, 2. procedure, 3. billing), 77% of obstetric hospitalizations and 30% of deaths met all three. Considering at least two of the three criteria, 82% of deaths were identified, while 11% were identified exclusively by diagnoses and 5% exclusively by procedures, as shown in Figures 1 and 2.

Of the 40,485 obstetric hospitalizations and 63 maternal deaths identified by criteria 2 and/or 3, but without diagnoses in Chapter XV, important participations of some diagnoses were observed as cause of hospitalization: Z30 (contraception), with 48.7% of hospitalizations and 12.6% of hospitalizations with death as outcome; P95 (fetal death of unspecified cause), with 13.7% of hospitalizations and 55.5% of hospitalizations with death as outcome; and I42 (cardiomyopathies), with 6.8% of hospitalizations and 4.7% of hospitalizations with death as outcome (Table 1 and Table 1 of supplementary material). Among the 63 deaths, 50 were related only to maternal procedure and 11 to procedure and delivery charge (Figure 2). The two remaining deaths were identified only by delivery charge (I50 heart failure and K65 peritonitis), with death of the pregnant woman and the fetus (Table 1 of supplementary material).

Among the 40,485 admissions, 27,971 met criteria 2 and 3 with 11 maternal deaths. From the point of view of delivery charging, 716 cases were discharge of the mother/postpartum woman with the newborn’s death and 3,859 were discharge of the mother/postpartum woman with fetal death. The P95 code was prevalent: 92.6% (663) of the 716 hospitalizations with newborn death and 97.7% (3,771) of the 3,859 hospitalizations with fetal death (Table 2 of supplementary material).

**Table 1.** Description of main diagnoses, codes, frequency, individual and cumulative representativeness of obstetric hospitalizations identified by procedures and maternal billing without diagnosis in chapter XV and other maternal codes in other chapters, Hospital Information System. Brazil, 2019.

Code	ICD-10 Code Description	Freq.	%	Cumulative %
Z30	Contraception (19.693 with code Z30.2 sterilization)	19,716	48.7	48.7
P95	Fetal death of unspecified cause	5,562	13.7	62.4
I42	Cardiomyopathies (2,419 with code I42.0 dilated cardiomyopathy)	2,766	6.8	69.3
N88	Other non-inflammatory disorders of the cervix (2092 with code N88.3 Cervical incompetence)	2,106	5.2	74.5
B24	Human immunodeficiency virus disease, unspecified	1,178	2.9	77.4
R10	Abdominal and pelvic pain	976	2.4	79.7
N39	Other urinary tract disorders	739	1.8	81.5
Z34	Normal pregnancy supervision	478	1.2	82.7
N93	Other abnormal bleeding from the uterus and vagina	426	1.0	83.7
Z35	Supervision of high-risk pregnancies	417	1.0	84.7
	too many codes	6,121	15.3	100.0
Total		40,485		

### Hospital Maternal Mortality Ratio, Maternal Mortality Ratio from the Adapted Mortality Information System and Maternal Mortality Ratio from the Total Mortality Information System

The total number of maternal deaths in the SIH amounted to 946 and, in the SIM, following the Ministry of Health rules, 1,576 were recorded in 2019, similar to what was disclosed by the MS/DATASUS^14^. However, in order to calculate the total SIM MMR, 1,575 maternal deaths were considered, resulting from the exclusion of the death of a 56-year-old woman who did not meet the inclusion criteria based on age.

**Figure 3.**
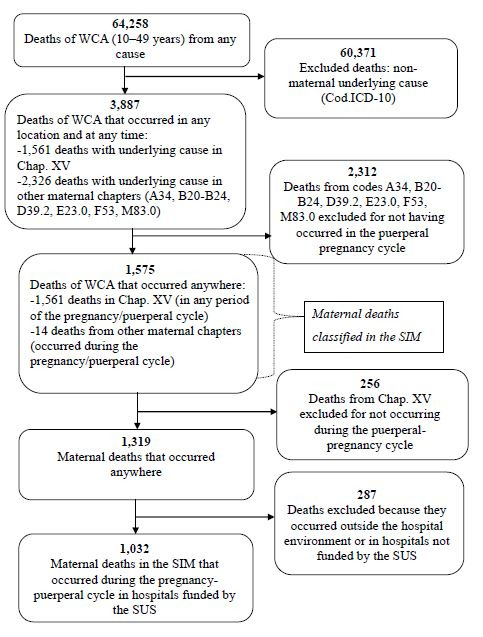
Flowchart of the steps for identifying maternal deaths that occurred during the pregnancy-puerperal cycle in hospitals funded by the Unified Health System, Mortality Information System. Brazil, 2019.

WCA: women in childbearing age; SIM: Mortality Information System; SUS: Unified Health System.

Figure 3 shows the step-by-step process for identifying the 1,032 deaths in the SIM (1,024 of Chapter XV and eight of the other Chapters—A34, B20-24, D39.2, E23.0, F53, M83.0) occurred during the pregnancy-puerperium cycle (up to 42 days after end of pregnancy) in SUS and affiliated hospitals.

For the calculation of MMR, the number of LB collected at SINASC in 2019 was 2,849,146, occurring in any location, a number used in the calculation of the total SIM MMR. To calculate the hospital MMR and adapted SIM MMR, the denominator was 2,077,630 LB in hospitals affiliated with the SUS.

Table 2 shows that the difference between hospital MMR and SIM MMR adapted for each 100,000 LB was 4.1 more maternal deaths recorded in SIM compared to SIH (p-value 0.053). The MMR increased with age, regardless of the cause of maternal deaths. The 30–39 age group stands out, with a difference in MMR between both databases (absolute difference 12.3, p=0.008), with greater coverage in the SIM. The MMR by region shows a difference between the databases in the Midwest (p-value 0.011) and in the South (p-value 0.002). The number of deaths and births to calculate the MMR is shown in Table 3 of the supplementary material. Figures 1 and 2 of the supplementary material show the MMR by age and region in graphic format.

**Table 2.** Maternal Mortality Ratio of deaths that occurred in hospitals affiliated with the Unified Health System, with data from the Hospital Information System and the adapted Mortality Information System, by age groups and regions. Brazil, 2019.

		MMR per 100 thousand LB	Absolute Difference: SIH/SIM	Relative difference: SIH/SIM	p-value
Global	SIH	45.5 (42.7−48.5)	-4.1 (-8.3−0.1)	0.9 (0.8−1.0)	0.053
	SIM*	49.7 (46.7−52.8)			
Age group (years)					
10–19	SIH	32.9 (27.3−39.4)	-6.0 (-14.8−2.7)	0.9 (0.7−1.1)	0.175
	SIM*	39.0 (32.8−45.9)			
20–29	SIH	38.3 (34.7−42.2)	1.0 (-4.2−6.3)	1.0 (0.9−1.2)	0.698
	SIM*	37.3 (33.7−41.1)			
30–39	SIH	57.1 (51.1−63.5)	-12.3 (-21.4−-3.3)	0.8 (0.7−1.0)	0.008
	SIM*	69.4 (62.8−76.4)			
40–49	SIH	138.2 (109.8−171.8)	-3.4 (-46.2−39.4)	1.0 (0.7−1.3)	0.876
	SIM*	141.6 (112.8−175.6)			
Region					
Mid-west	SIH	46.3 (35.6−59.2)	-23.5 (-41.6−-5.4)	0.7 (0.5−0.9)	0.011
	SIM*	69.8 (56.5−85.3)			
Northeast	SIH	51.4 (45.9−57.2)	3.9 (-3.8−11.7)	1.1 (0.9−1.3)	0.319
	SIM*	47.4 (42.2−53.1)			
North	SIH	57.5 (48.7−67.5)	-5.3 (-18.6−7.9)	0.9 (0.7−1.2)	0.432
	SIM*	62.9 (53.7−73.3)			
Southeast	SIH	45.1 (40.4−50.1)	-3.3 (-10.2−3.6)	0.9 (0.8−1.1)	0.348
	SIM*	48.4 (43.5−53.6)			
South	SIH	22.6 (17.5−28.8)	-13.9 (-22.8−-5.0)	0.6 (0.5−0.9)	0.002
	SIM*	36.5 (29.9−44.2)			

MMR: Maternal Mortality Ratio; LB: live births; SIH: Hospital Information System; SIM: Mortality Information System. *Adapted SIM: maternal deaths that occurred in hospitals or health facilities funded by the SUS. SIM-SUS, 2019.

Finally, the overall SIM MMR calculated in this study was 55.3 per 100,000 LB (1,575/2,849,146 *100,000 LB). It differs from the Ministry of Health^15^ RMM of 57.9, corrected for the correction factor.

## DISCUSSION

In Brazil, where 98.5% of births take place in hospitals^16^, the availability of a national hospital database can contribute to the study of MM by extrapolating its reimbursement function, bringing data on 2,497,957 obstetric hospitalizations and 946 maternal deaths in 2019.

Understanding MM with the help of the SIH as complementary to the SIM, being able to provide deaths that would not usually be registered, can be an alternative to revert the current situation of difficult reduction of MMR, especially when the temporality of MMR shows stagnation and growth, as in Brazil^17,18^ and in the United States^19,20^.

Both maternal mortality and morbidity result from the difficulty or lack of access to medical services, social, economic and demographic conditions that impact the risk of developing complications during and after pregnancy^21,22^. Studies on severe maternal morbidity demonstrate how the SIH serves to identify near-death cases of WCA, speeding up recognition, compared to studies based on medical records and interviews^23,24^.

We identified 40,485 hospitalizations and 63 deaths that would not be recognized as they did not contain maternal patterns among the hospitalization causes. These findings make up 1.6% of all obstetric admissions (2,497,957) and 6.7% of all admissions with death as outcome (946). With the use of procedure and billing criteria, we found incorrect use of diagnosis codes (Table 1 and Table 1 of supplementary material), with a significant presence of codes Z30, I42 and the pediatric code P95. We suggest that the use of contraception codes (Z30) and fetal death (P95) be reassessed for a possible change of code, accompanied by guidance to the person filling out the Hospital Admission Authorization. As for the code for cardiomyopathies (I42), we suggest a more in-depth study on its use. The use and correct completion of billing variables can help in investigations of fetal and perinatal death by exposing the hospital trajectory of the WCA (Table 1 of supplementary material).

The hospital MMR obtained in this study was 45.5 (95% confidence interval [CI] 42.7–48.5) per 100,000 LB in 2019, which reflects the difficulty of reaching a maximum MMR of 30 by 2030, according to the Sustainable Development Goals (SDG)^17^.

When we compared maternal deaths from SIM adapted MMR (n=1,032), 92% were also found in the SIH (n=946), with the limitation of assessing aggregated data and not necessarily being the same women in both databases. Our findings comparing the regions were interesting, given that sometimes the MMR was higher in the SIM, sometimes in the SIH. Regions where the SIM adapted MMR was greater than that estimated by the SIH could suggest that the investigation of maternal deaths has been carried out more effectively, increasing deaths in the SIM after review. This is what we assume to have happened in the South and Midwest MMR. According to a study^17^, the regions with the highest percentages of deaths of WCA investigated in 2017 were the South, followed by the Midwest and Southeast. The opposite occurred in the Northeast, where we found more deaths in the SIH than in the adapted SIM. Although it is an uncertain estimate (absolute difference 3.9, 95%CI -3.8−11.7 per 100,000 LB), this finding can be explored in other years and, upon confirmation, be investigated.

Another possible explanation for the difference could be related to hospitals with mixed obstetric beds, with part of the beds linked to SUS and part of beds being supplementary/private. As one of the selection criteria for comparable maternal deaths of SIM and SIH came from the hospitals where all obstetric admissions of the SIH occurred, there may have been a selection of maternal deaths in the SIM in a health establishment common to both bases, but in beds not affiliated with SUS and, therefore, not present in the SIH database.

Exposing the known increased risk of MM with advancing age, the hospital MMR of the age group of 40–49 years was three times greater than the overall MMR of the SIH and four times greater than the age group of 10–19 years, showing that the MMR of women over 40 years old still remains high, even though the literature points to significant reductions in the MMR of women aged 40–49 years in Brazil from 1996 to 2018^18,25^.

Completing the following SIH variables would be useful in maternal studies:

1. “Gestrisco”—high-risk pregnancy;
2. “Insc_pn”—enrollment of the pregnant woman in the prenatal assistance program;
3. “Num_filhos”—number of children. The “insc_pn” variable would allow, through record linkage techniques, the crosschecking with other databases, even without nominal data^26^. Information on number of children could indicate that primiparous or multiparous women are a maternal risk factor.

We reinforce the importance of training in proper filling out of the Hospital Admission Authorization, normally carried out by an administrative professional who is not trained to extract data from medical records, aiming at billing and reimbursement by the SUS, often making the diagnosis and procedure compatible to avoid reimbursement glosses^27,28^.

The unit of analysis being the Hospital Admission Authorization is a limitation. Without access to nominal data, possible records of rehospitalization of the same patient were not recognized, which we assume occurs mainly in postpartum hospitalizations. Another limitation is the possible risk of information bias inherent in the use of secondary data.

One limitation of this study was the failure to stratify the calculation of the SIH and SIM MMR by race/color, given their relevance in exposing maternal health racial inequalities. The inherent complexity of the topic includes missing data that are unlikely to be distributed completely by chance^29^.

Traditionally, the measurement of maternal deaths in health databases uses the ICD-10 codes. This study identified a larger contingent of hospitalizations and deaths with the inclusion of variables procedures performed and delivery charges from the SIH. Although there are differences between hospital MMR and adapted SIM MMR, the use of the SIH as a complementary information system can be valid in research on maternal mortality and morbidity.
