# Supplementary material for: Usefulness of the Hospital Information System for maternal mortality surveillance in Brazil
Source: Rev Bras Epidemiol. 2023 Jan 9;26:e230007. doi: 10.1590/1980-549720230007.2 (PMC9838231; doi:10.1590/1980-549720230007.2)

MATERIAL SUPLEMENTAR:

Tabela 1: Descrição dos diagnósticos principais (códigos, frequência, representatividade individual), dos procedimentos e da cobrança das internações obstétricas com óbito identificadas pelos procedimentos e cobrança materna sem diagnóstico do capítulo XV e demais códigos maternos de outros capítulos, SIH. Brasil, 2019.

Cód.	Descrição Código CID-10	Freq.	%	Procedimento	Cobrança materna
P95	Morte fetal de causa não especificada	35	55,5	20 Parto normal<br>7 Parto cesariano<br>5 Parto cesariano em gestante de alto risco<br>2 Parto normal em gestante de alto risco e ou eclampsia<br>1 Tratamento de intercorrências clínicas na gravidez	4 Óbito da gestante e do concepto
Z30	Anticoncepção	8	12,6	Parto cesariano com laqueadura tubária com diagnósticos de esterilização	4 Óbito mãe /puérpera/alta RN<br>1 Óbito mãe/puérpera/perman. RN
I42	Cardiomiopatias	3	4,7	Tratamento de intercorrências clínicas na gravidez	
A41	Outras septicemias	1	1,6	Curetagem pós- abortamento/puerperal	Óbito da gestante e do concepto
B24	Doença pelo vírus da imunodeficiência humana NE	1	1,6	Curetagem pós- abortamento/puerperal	
D50	Anemia por deficiência de ferro	1	1,6	Tratamento de intercorrências clínicas na gravidez	
G83	Outras síndromes paralíticas	1	1,6	Tratamento de complicações relacionadas predominantemente ao puerpério	
I27	Outras formas de doença cardíaca pulmonar	1	1,6	Parto normal em gestante de alto risco e ou eclampsia	Óbito da gestante e do concepto
*I50**	*Insuficiência cardíaca*	*1*	*1,6*	*Tratamento de insuficiencia cardíaca (303060212)*	*Óbito da gestante e do concepto*
I51	Complicações cardiopatias/doenças cardíacas mal def.	1	1,6	Parto normal em gestante de alto risco e ou eclampsia	
J11	Influenza devida a vírus nao identificado	1	1,6	Parto cesariano em gestante de alto risco	
J18	Pneumonia por microorganismo NE	1	1,6	Tratamento de intercorrências clínicas na gravidez	
J96	Insuficiência respiratória não classificada outra parte	1	1,6	Parto normal em gestante de alto risco e ou eclampsia	
*K65**	*Peritonite*	*1*	*1,6*	*Tratamento de doenças do peritônio (303070080)*	*Óbito da gestante e do concepto*
N11	Nefrite tubulo-intersticial crônica	1	1,6	Parto cesariano em gestante de alto risco	
N17	Insuficiência renal aguda	1	1,6	Tratamento de complicações relacionadas predominantemente ao puerpério	
Q00	Anencefalia e malformações similares	1	1,6	Curetagem pós- abortamento/puerperal	
R00	Anormalidades do batimento cardíaco	1	1,6	Curetagem pós- abortamento/puerperal	
R99	Outras causas mal definidas e as NE	1	1,6	Parto cesariano	
Z60	Problemas relacionados com o meio social	1	1,6	Parto cesariano com laqueadura tubária com diagnósticos de esterilização	
	**Total**	**63**	**100**		

*Óbitos maternos identificados pela cobrança de parto, sem diagnóstico e/ou procedimento materno

Tabela 2: Descrição das internações de MIF identificadas pelos procedimentos e cobrança materna sem diagnóstico do capítulo XV e demais códigos maternos de outros capítulos, SIH. Brasil, 2019.

	**Cobrança materna**							
**Procedimentos Maternos**	Alta mãe /puérpera e RN	Alta mãe /puérpera e permanência RN	Alta mãe/puérpera e óbito RN	Alta mãe /puérpera com óbito fetal	Óbito da gestante e do concepto	Óbito mãe /puérpera e alta RN	Óbito mãe /puérpera e permanência RN	**Total**
Parto cesariano com laqueadura tubária com diagnósticos de esterilização	17.879	1.041	20	37	0	4	1	18.982
Parto normal	945	45	495	2.702	1	0	0	4.188
Parto cesariano	1.647	123	152	664	3	0	0	2.589
Parto cesariano em gestante de alto risco	1.248	202	26	186	0	0	0	1.662
Parto normal em gestante de alto risco e ou eclampsia	211	34	15	235	1	0	0	496
Parto normal em centro de parto normal (CPN)	9	1	8	35	0	0	0	53
Curetagem pós-abortamento/puerperal	0	0	0	0	1	0	0	1
**Total**	21.939	1.446	716	3.859	6	4	1	27.971

Tabela 3: Óbitos e nascimentos ocorridos em hospitais conveniados ao SUS, com os dados do SIH e SIM, por grupos etários e por regiões. Brasil, 2019.

		**Óbitos**	**NV***
**Global**	**SIH**	946	2.077.630
	**SIM****	1.032	2.077.630
**Grupo etário (anos)**			
**10 - 19**	**SIH**	120	364.348
	**SIM****	142	364.348
**20 - 29**	**SIH**	407	1.062.219
	**SIM****	396	1.062.219
**30 - 39**	**SIH**	338	592.453
	**SIM****	411	592.453
**40 - 49**	**SIH**	81	58.610
	**SIM****	83	58.610
**Região da residência**			
**CO**	**SIH**	63	136.112
	**SIM****	95	136.112
**NE**	**SIH**	326	634.837
	**SIM****	301	634.837
**N**	**SIH**	151	262.382
	**SIM****	165	262.382
**SE**	**SIH**	341	756.894
	**SIM****	366	756.894
**S**	**SIH**	65	287.405
	**SIM****	105	287.405

*Nascidos vivos nos hospitais conveniados ao SUS. SINASC-SUS, 2019.

** SIM adaptado: óbitos maternos ocorridos em hospitais financiados pelo SUS. SIM-SUS,2019.

Figura 1: Razão de Mortalidade Materna com os dados do SIH e SIM adaptado, por grupos etários. Brasil, 2019.

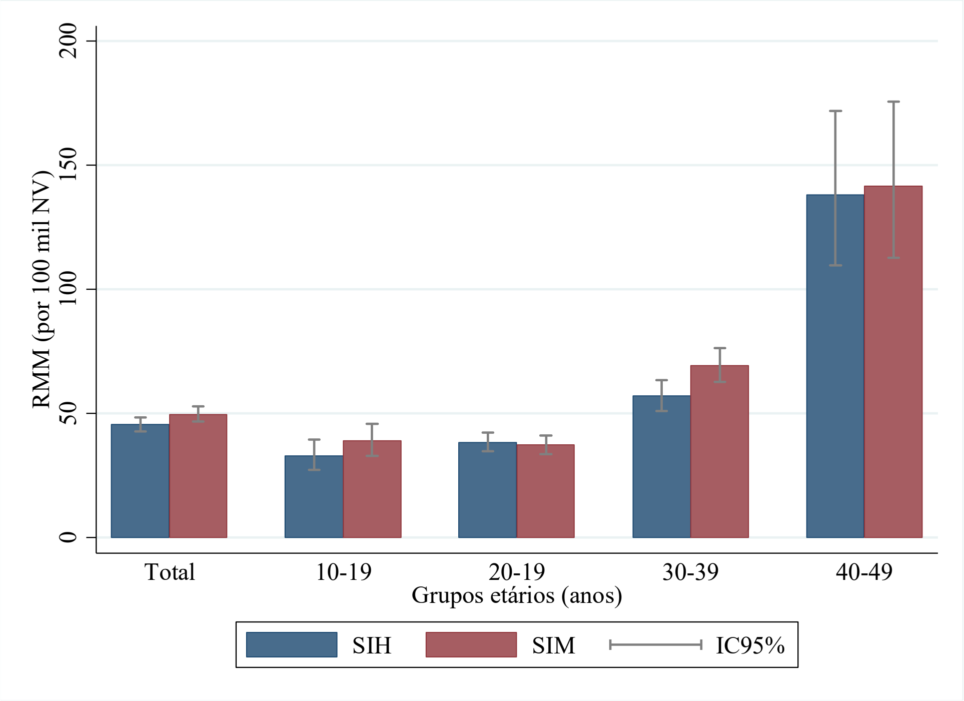

Figura 2: Razão de Mortalidade Materna com os dados do SIH e SIM adaptado, por regiões. Brasil, 2019.

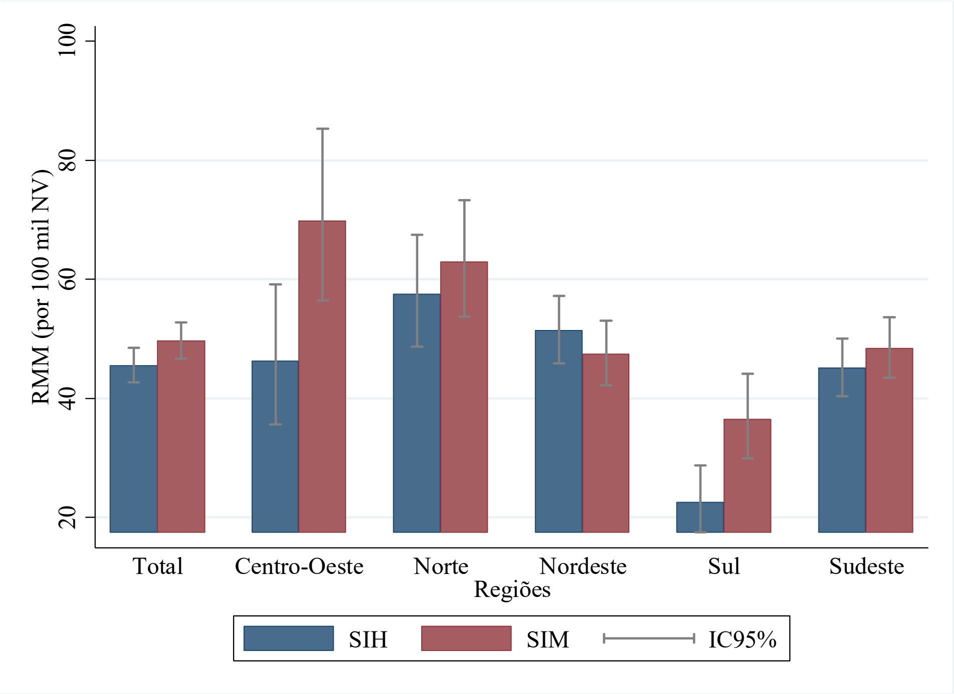

Supplement: Supplementary file 1 [file 1980-5497-rbepid-26-e230007-s1.pdf]
